# BiocPkgTools: Toolkit for mining the *Bioconductor* package ecosystem

**DOI:** 10.12688/f1000research.19410.1

**Published:** 2019-05-29

**Authors:** Shian Su, Vincent J. Carey, Lori Shepherd, Matthew Ritchie, Martin T. Morgan, Sean Davis

**Affiliations:** 1 Molecular Medicine Division, The Walter and Eliza Hall Institute of Medical Research, Parkville, Australia; 2 Channing Division of Network Medicine, Brigham and Women’s Hospital, Boston, MA, USA; 3 Roswell Park Cancer Institute, Buffalo, NY, USA; 4 Center for Cancer Reasearch, National Cancer Institute, National Institutes of Health, Bethesda, MD, USA

**Keywords:** bioinformatics, r, bioconductor, software, reproducible research

## Abstract

**Motivation:** The Bioconductor project, a large collection of open source software for the comprehension of large-scale biological data, continues to grow with new packages added each week, motivating the development of software tools focused on exposing package metadata to developers and users. The resulting BiocPkgTools package facilitates access to extensive metadata in computable form covering the Bioconductor package ecosystem, facilitating downstream applications such as custom reporting, data and text mining of Bioconductor package text descriptions, graph analytics over package dependencies, and custom search approaches.

**Results: **The BiocPkgTools package has been incorporated into the Bioconductor project, installs using standard procedures, and runs on any system supporting R. It provides functions to load detailed package metadata, longitudinal package download statistics, package dependencies, and Bioconductor build reports, all in "tidy data" form. BiocPkgTools can convert from tidy data structures to graph structures, enabling graph-based analytics and visualization. An end-user-friendly graphical package explorer aids in task-centric package discovery. Full documentation and example use cases are included.

**Availability: **The BiocPkgTools software and complete documentation are available from Bioconductor ( https://bioconductor.org/packages/BiocPkgTools).

## Introduction

*Bioconductor* is a open source software project (comprising 1741 individual analysis packages) and community for the analysis and comprehension of large-scale biological data. Newly submitted software packages undergo a technical review to ensure that best practices and *Bioconductor* coding conventions are followed. The project maintains an automated build system that ensures that packages in the *Bioconductor* project are compiled and built successfully and pass basic checks. Package downloads are tracked and aggregated by package and month, longitudinally. Finally, package details such as title, description, version, author, and dependencies on other R packages are compiled based on package metadata.

The current size and growth of the *Bioconductor* project suggests that there is merit in exposing computable forms of the metadata describing the *Bioconductor* package ecosystem. To that end, we developed a small suite of tools, BiocPkgTools, to provide easy access to project details such as download statistics, bulk package metadata, and package build status. Developers, project leaders, open source software researchers, and *Bioconductor* end users can build on the availability of these data for applications such as custom reporting, dependency graph analytics, package filtering, and text mining.

## Features and usage

**Figure 1.**
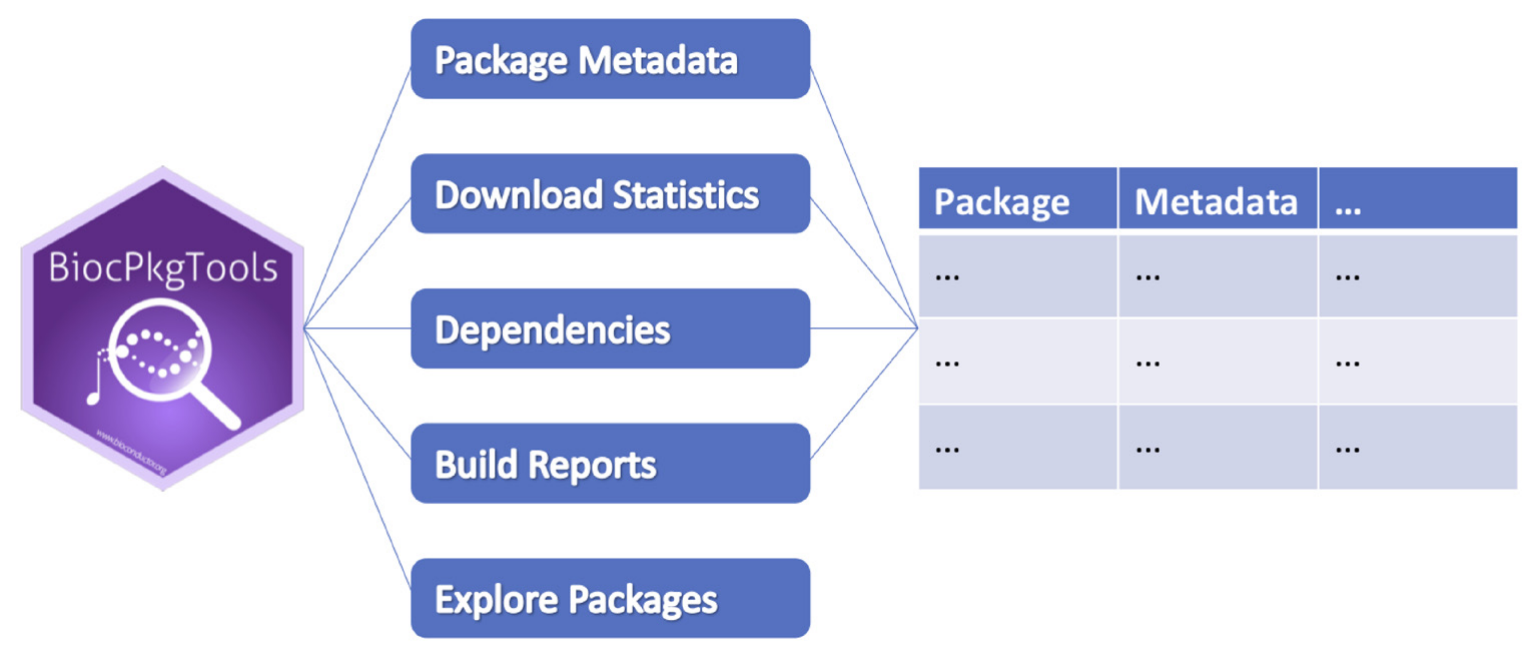
Schematic overview of the BiocPkgtools package. BiocPkgTools can access and transform web-accessible resources including package metadata, download statistics, dependencies between packages, and updated *Bioconductor* build report status to "tidy data" reports that can be manipulated using standard R tools. Interactive package exploration is also available.

The core functionality of BiocPkgTools is to expose *Bioconductor* project and package metadata as tidy data ^1^ objects ( Figure 1). The data presented by the package are accessed directly from online resources available from *Bioconductor*. As such, the package relies on web connectivity and collects the most recent data. Installation instructions are detailed on the package website.

Package functionality can be roughly divided into data access, data presentation, and graph/network functionality. See Table 1 for an overview.

After installing BiocPkgTools, the biocDownloadStats function can generate a tidy data structure summarizing monthly download statistics (both total and unique IP addresses) for all *Bioconductor* packages.


library(BiocPkgTools)                                                 
dlstats = biocDownloadStats()                                         
head(dlstats, 3)                                                      

## # A tibble: 3 x 6                                                  
##   <fct>   <int> <fct>              <int>           <int> <chr>     
## 1 ABarray  2018 Jan                  117             150 Software  
## 2 ABarray  2018 Feb                   97             125 Software  
## 3 ABarray  2018 Mar                  102             121 Software  


**Table 1.** Main package functions and descriptions.

Name	Functionality
biocPkgList	Package details including description, author and maintainer, dependencies, URLs, bug report mechanism
biocDownloadStats	Monthly download statistics for all packages
biocbuildReport	*Bioconductor* build report for all packages and systems
biocExplore	Interactive, browsable “bubble plot” of *Bioconductor* packages and details
problemPage	Interactive, customized build report for an individual package author
buildPkgDependencyDataFrame	Package dependencies as data frame
buildPkgDependencyIgraph	Package dependencies as a graph ^2^
inducedSubgraphByPkgs	Create a minimal subgraph of *Bioconductor* dependencies based on specific packages
subgraphByDegree	Create a subgraph of all packages within a given degree of a single package

The biocBuildReport function gathers information from the *Bioconductor* build report site and can be used, for example, to summarize the “build status” for all *Bioconductor* pacakages.


buildrep = biocBuildReport(version = "3.9")       
table(buildrep$stage, buildrep$result)            

##                                                
##           ERROR   OK skipped TIMEOUT WARNINGS  
##  buildbin     2 3352      70       0        0  
##  buildsrc    93 5057       0       5        0  
##  checksrc    57 4181      98       8      811  
##  install     39 5116       0       0        0  


These data are useful to developers to track the health of their software either programmatically or via a searchable, sortable table from the problemPage function.

**Figure 2.**
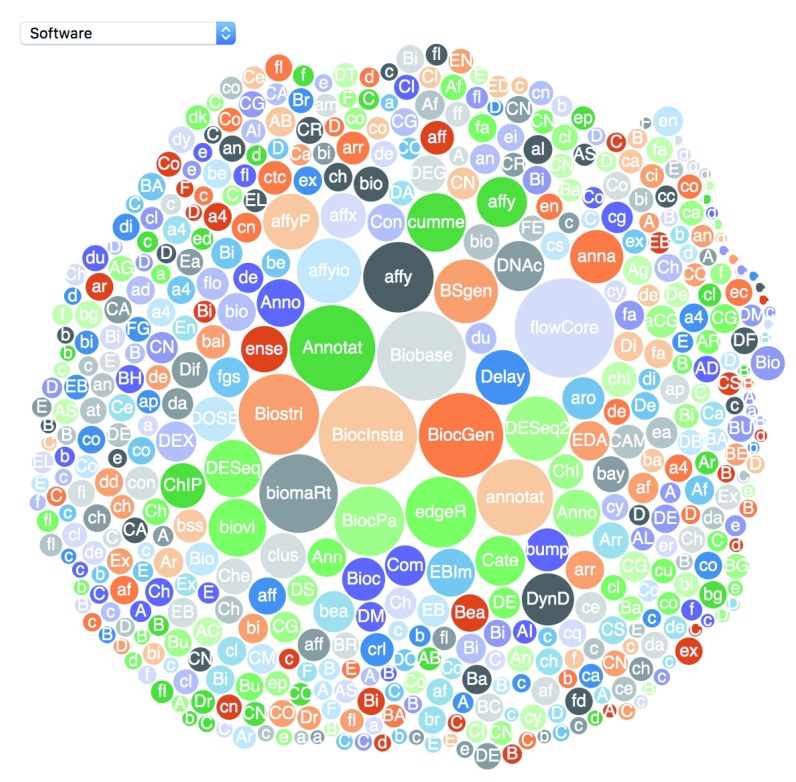
The biocExplore function opens an interactive web application that allows users to select focused groups of *Bioconductor* packages to view as a bubble plot. Bubbles are sized based on download statistics. Hovering over a bubble will give download number while clicking on a bubble will pop up a package details page, including a link to the package landing page.

As an alternative to basic web browser search and the *Bioconductor* online software list, the biocExplore function offers interactive and graphical approach to package browsing (see Figure 2). The biocExplore widget allows browsing packages under different biocViews, Bioconductor’s software catergory tags. This interactively visualises the relative number of downloads each package has under different biocViews, allowing users to quickly determine which packages are most commonly used for different analysis tasks.

**Figure 3.**
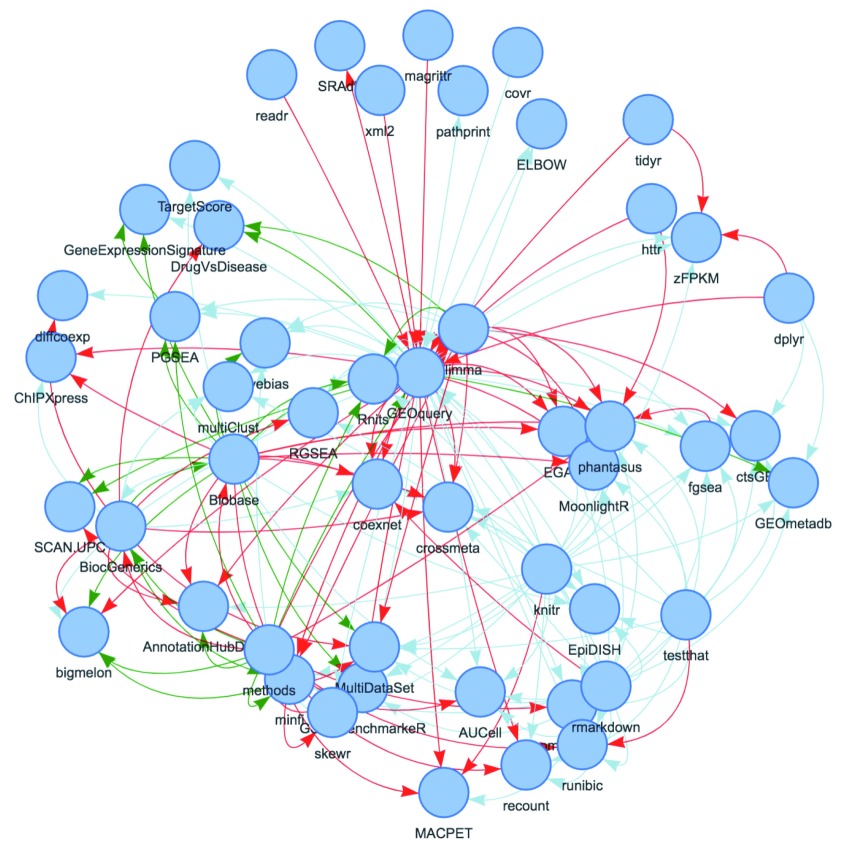
The subgraphByDegree function builds a data visualization of dependencies between all packages within one degree of the GEOquery package using the visNetwork package ^3^. Links are colored based on type (Suggests [light blue], Depends [green], and Imports [red]) and arrows point to the “dependent” package.

The *Bioconductor* package ecosystem is, by design, highly interconnected via package dependencies. Several functions in the BiocPkgTools package provide examples of package dependency graph creation and visualization. Figure 3 displays packages within one degree of dependency relationship of the GEOquery package.

### Implementation

BiocPkgTools is implemented as a standard R package and hosted in the *Bioconductor* repository. All functions are documented and include examples. An included tutorial (vignette) demonstrates features and capabilities.

## Discussion

The BiocPkgTools package comprises a set of functions for accessing software metadata from the growing collection of *Bioconductor* packages. For software developers, this metadata can be useful for tracking package build status and the health of package dependencies. Easy access to descriptive package metadata for all *Bioconductor* software resources can empower researchers or users interested in text mining, custom package search, or analysis of the existing software ecosystem. BiocPkgTools can provide easy access to metrics of *Bioconductor* sofware usage that are increasingly being incorporated into funding and promotion decisions.

## Data availability

All data accessed and used by the BiocPkgTools package are publicly available and are updated regularly at the *Bioconductor* project.

## Software availability

- Software available from: https://bioconductor.org/packages/BiocPkgTools
- Source code available from: https://github.com/seandavi/BiocPkgTools
- Archived source code as at time of publication: https://doi.org/doi:10.18129/B9.bioc.BiocPkgTools ^4^
- License: MIT License
