# Supplementary material for: BiocPkgTools: Toolkit for mining the Bioconductor package ecosystem
Source: F1000Res. 2019 May 29;8:752. [Version 1] doi: 10.12688/f1000research.19410.1 (PMC6584971; doi:10.12688/f1000research.19410.1)
Supplement: Peer review history

# Peer review: Reviewer response for version 1

**DOI:** 10.5256/f1000research.21277.r49217

**Published:** 2019-06-21

**Authors:** Henrik Bengtsson

**Affiliations:** 1 Division of Bioinformatics, Department of Epidemiology and Biostatistics, University of California, San Francisco, San Francisco, CA, USA

This article presents the BiocPkgTools package, which provides an R API to the various package metadata that is available mostly in human-readable formats on the https://bioconductor.org/ website. By providing an R API for accessing package metadata, the authors argue that "data mining and value-added functionality such as package searching, text mining, and analytics on packages" may follow.

 I believe that this package will add value to the Bioconductor community and beyond. It is likely that the package will lower the threshold for doing "data mining" on Bioconductor packages. The reports that are based on these metadata and produced by this package are likely to inspire others to produce other types of reports and interactive tools.

 Having said, I do think there is room for some immediate improvements to the article, which might also spill over to the package itself.

**Major:**

 The role of the package:

- It is not clear whether this package is to be considered a Bioconductor core package or a user-contributed package. That can only be inferred/guessed from authors list and possible from the package name.
- If it is an official core Bioconductor package (similar to BiocManager) supported and maintained by the Bioconductor Team, then I think it would be of value to make that explicit. This would change the expectation on the package and its long-term support, e.g. will it break or not when the Bioconductor website changes, what should be documented as part of the package and what should be documented on the Bioconductor website., etc. The questions in the following section illustrate this.

Description on the metadata:

- There is no explicit reference to the source data, e.g. is this package using a public or a private Bioconductor Online API to gather the data. Is this API, or URLs official and stable, or is this package meant to play that role?
- Is there another reference from where one can learn more about how the data is collected? Are data from other Bioconductor mirrors included in the download stats?
- The download stats do not contain information on the package version, which is mentioned in the package vignette. However, it's not clear whether it is only downloads from the current release branch that are counted or not. For instance, if I download a package for a legacy Bioconductor release version or the current developer version, will that add to the data?

**Minor/trivial:**

 API:

- Given the package title 'Collection of simple tools for learning about Bioc Packages' and description, the `biocBuildEmail()` function seems to be an odd-one-out.

Typos/spelling:

- Section 'Introduction': "a open source software" -> "an open-source software".
- Section 'Features and usage': missing "the" in "to expose [the] Bioconductor project".
- Section 'Features and usage': "software cate[r]gory tags".
- Section 'Discussion': ... Bioconductor sof[t]ware...
- Table 1: biocbuildReport -> biocBuildReport.
- Table 1 caption: "all Bioconductor pac[a]kages".
- Grammar in Section 'Introduction': Should "suggest" be used instead of "suggests" in "The current size and growth of the Bioconductor project suggests"?
- In addition, `spelling::spell_check_package()` on the package itself reveals several mistakes.
- Nomenclature: Bioconductor is sometimes referred to as 'bioconductor' (lower case) or just 'bioc' and 'Bioc' (the latter is even used in the package title).

Figures and tables:

- Figure 1: It's not clear what the different parts of this figure are. As I understand it, the left part (the hex logo) represents the package itself, and the middle part (five blocks) represents the "web-accessible resources" that the package queries.  What does the table and the edges in the right part represent?  Is it meant to illustrate that the package pulls data from 5 different sources and joins them into a single table?  The caption also tries to mention "Interactive package exploration is also available" which makes it harder to understand the figure.  Maybe it's the ordering of these three parts are confusing.  Maybe it would be more clear if the edges from the hex logo to the web resources would be dropped.  Maybe it would help if the empty table could be populated with something else than just "..." entries.  Alternative, maybe something like this would clarify the flow of the data?

Package/examples:

- Section 'Implementation': "All functions are documented and include examples." Maybe rephrase so it doesn't sound that there are examples for *all* functions, which is not the case.
- Coding Convention: The package vignette and examples, and the example code snippets in the article, use `a = b` for assignments rather than `a <- b` that is the recommended coding style for Bioconductor, df. https://www.bioconductor.org/developers/how-to/coding-style/.
- EXAMPLES: The presented output of the `dlstats = biocDownloadStats()` example is outdated; it shows other fields/columns that what the current BiocPkgTools 1.2.0 outputs.
- In addition to the above, I've identified other package-specific issues that I reported directly to the package's issue tracker ( https://github.com/seandavi/BiocPkgTools/issues/34).

I confirm that I have read this submission and believe that I have an appropriate level of expertise to confirm that it is of an acceptable scientific standard, however I have significant reservations, as outlined above.

# Peer review: Reviewer response for version 1

**DOI:** 10.5256/f1000research.21277.r49235

**Published:** 2019-06-19

**Authors:** Mike Smith

**Affiliations:** 1 Genome Biology Unit, European Molecular Biology Laboratory, Heidelberg, Germany

In this paper Su et al present BiocPkgTools, an R package that provides programmatic access to metadata about software in the Bioconductor project. The package is available from Bioconductor and the source code can be easily viewed on Github. The metadata it can access includes download numbers, package 'health' status, topic categories, and software dependencies.

 Since the software forms at least as large a part of this publication as the article, I have tried to review both.

**Paper:**

 The paper is succinct and to the point. In some instances there is an implied understanding of R package development and the Bioconductor build system. For example, in the code block presented to demonstrate the biocBuildReport function, the row names e.g. buildbin, checksrc etc are probably fairly meaningless to someone unfamiliar with Bioconductor's continuous integration platform. It's fair to say most interested readers will already be Bioconductor package developers, but a caption linking this back to the build system discussion in the introduction would add clarity.

 Similarly the biocViews concept is not full described, but forms a key part of one the packages main functions: biocExplore(). Expanding a little on how these terms are assigned to packages (i.e. mostly but not always by the package authors) might make it clearer to users why some values return unexpected results e.g. 'Bioinformatics' only shows me 15 packages presumably because most package authors see this as redundant, although 'Software' feels similarly obvious yet returns a huge number of packages as this is assigned by Bioconductor itself.

 The motivation behind providing programmatic access to build reports and download statistics is presented in the text. However, it would be nice for the authors to expand upon what they feel the use cases for the package dependency graphs may be. They look cool, but based on the paper content it's not immediately obvious to me where they might be used. The discussion highlights the fact that download metrics etc are gaining traction as a measure factored into funding decisions, and one thing that springs to mind is that a similar point can be made visually for software infrastructure that has many downstream dependents. My efforts to create a `subgraphByDegree()` with degree greater than one (to view a larger software stack) creates a graph to large to render, so an example of how to visualise this (i.e. show all downstream dependsOnMe packages in a tree) would be a great addition to either the paper or the package vignette.  

**Minor points:**

 I recommend providing a link to the BiocPkgTools landing page after the sentence "Installation instructions are detailed on the package website."

 Indicate that the code to produce Figure 3 can be found in the package vignette (or include it as supplementary material here).  Since it is quite a bit more than a one-liner to produce Figure 3 I think it would be beneficial to point readers to the code.

 Perhaps I am mistaken, but I can find no mention in the paper or package of what the colours represent in the bubble plots produced by biocExplore(). They definitely feel like they should mean something.

 I would consider rephrasing the sentence "This interactively visualises the relative number of downloads each package has under different biocViews".  To me this reads like the plot is somehow visualising how relevant the package is to the selected view, when really it is just filtering the package list based on the biocView category.

 Typos (given in context, specific [word] highlighted):

- for all Bioconductor [pacakages]
- software [catergory] tags
- Bioconductor [sofware] usage
- bioc[b]uildReport (Table 1)

 

**Software:**

 In general the software is well written with a comprehensive vignette providing more extensive examples than seen in the paper. However I did encounter a few issues when testing aspects of the package:

- The function firstInBioc() fails with 'Error in desc(Date) : could not find function "desc"'.  Similarly, the function inducedSubgraphByPkgs() fails with 'Error in V(g2) <- `*vtmp*` : could not find function "V<-"' 
  - I think in both cases the relevant functions need to be added as imports in the NAMESPACE.  These work in the package examples as additional libraries are loaded first.
- The manual page for subgraphByDegree() is slightly confusing.  The argument pkg takes a vector of length one, but the description field reads like multiple packages can be supplied.
- Why is the function get_bioc_data() in snake_case, while all other functions are named with camelCase?
- I wonder if the function biocBuildEmail() should really be exported.  This seems very specific to the Bioconductor core team, but is very similarly named to biocBuildReport() which is one of the primary data getters and will be widely used.  Also, the 'Usage' section of the biocBuildEmail function references the function .getTemplatePath() which is not exported, perhaps reflecting the niche nature of this function.
- It's not clear to me what the difference is between an 'induced subgraph' and a 'regular subgraph' in the functions inducedSubgraphByPkgs() vs subgraphByDegree() and I couldn't find an explanation in the package documentation. Perhaps this is just a lack of knowledge about graphs on my part, but it's information I looked for based on the different function names.
- Are all of the packages listed in the Suggests field of the DESCRIPTION file used? I can't find mention of tm, SnowballC

I confirm that I have read this submission and believe that I have an appropriate level of expertise to confirm that it is of an acceptable scientific standard.

# Peer review: Reviewer response for version 1

**DOI:** 10.5256/f1000research.21277.r49219

**Published:** 2019-06-10

**Authors:** Simina M. Boca

**Affiliations:** 1 Innovation Center for Biomedical Informatics, Department of Oncology, Georgetown University Medical Center, Washington, DC, USA

This article presents the new Bioconductor package, BiocPkgTools, which allows users to obtain various statistics about Bioconductor packages, including the number of downloads by month, the build status, and the package dependencies.

 Overall, this seems to be a very useful package! I'm excited about using it as a way of measuring the use of my own package and perhaps doing some other exploratory analyses on the Bioconductor ecosystem. I was able to load and run everything and obtain similar results to those presented (slight differences due to running it at a different time) after reading this article and the package vignette.

 I believe the following minor edits could improve the presentation:

1. Provide the code for generating Figures 2 & 3, even though it is in the vignette. This would be helpful from a reproducibility perspective.
2. Specify how often the data presented are updated. Presumably this is done almost immediately, given that the build status is included. It would also be interesting to give an idea of how this is done.
3. Specify in the output/help page that the filtering in the bubble chart obtained by running biocExplore() is done by biocViews (this can be a bit confusing since, for example, under "Bioinformatics" only a few packages are selected, but that is not something you can fix, since it's up to the authors to include the appropriate biocViews).
4. Specify that the stats downloaded from biocDownloadStats() are the total number of downloads, not the number of downloads from distinct IPs, as the Bioconductor package page provides both.

I confirm that I have read this submission and believe that I have an appropriate level of expertise to confirm that it is of an acceptable scientific standard.
